# Vaccinations and childhood type 1 diabetes mellitus: a meta-analysis of observational studies

**DOI:** 10.1007/s00125-015-3800-8

**Published:** 2015-11-12

**Authors:** Eileen Morgan, Sophia R. Halliday, Gemma R. Campbell, Chris R. Cardwell, Chris C. Patterson

**Affiliations:** UKCRC Centre of Excellence for Public Health NI, Queen’s University Belfast, Belfast, UK; Centre for Public Health, Queen’s University Belfast, Grosvenor Road, Belfast, BT12 6BJ UK; Centre for Statistical Science and Operational Research, Queen’s University Belfast, Belfast, UK

**Keywords:** Epidemiology, Meta-analysis, Type 1 diabetes, Vaccinations

## Abstract

**Aims/hypothesis:**

The aim of this study was to investigate the association between routine vaccinations and the risk of childhood type 1 diabetes mellitus by systematically reviewing the published literature and performing meta-analyses where possible.

**Methods:**

A comprehensive literature search was performed of MEDLINE and EMBASE to identify all studies that compared vaccination rates in children who subsequently developed type 1 diabetes mellitus and in control children. ORs and 95% CIs were obtained from published reports or derived from individual patient data and then combined using a random effects meta-analysis.

**Results:**

In total, 23 studies investigating 16 vaccinations met the inclusion criteria. Eleven of these contributed to meta-analyses which included data from between 359 and 11,828 childhood diabetes cases. Overall, there was no evidence to suggest an association between any of the childhood vaccinations investigated and type 1 diabetes mellitus. The pooled ORs ranged from 0.58 (95% CI 0.24, 1.40) for the measles, mumps and rubella (MMR) vaccination in five studies up to 1.04 (95% CI 0.94, 1.14) for the haemophilus influenza B (HiB) vaccination in 11 studies. Significant heterogeneity was present in most of the pooled analyses, but was markedly reduced when analyses were restricted to study reports with high methodology quality scores. Neither this restriction by quality nor the original authors’ adjustments for potential confounding made a substantial difference to the pooled ORs.

**Conclusions/interpretation:**

This study provides no evidence of an association between routine vaccinations and childhood type 1 diabetes.

**Electronic supplementary material:**

The online version of this article (doi:10.1007/s00125-015-3800-8) contains peer-reviewed but unedited supplementary material, which is available to authorised users.

## Introduction

Despite much research attempting to identify perinatal risk factors of childhood type 1 diabetes mellitus, the environmental factors thought to trigger autoimmune destruction of the insulin-producing cells in the pancreas remain largely unknown [1]. An increasing incidence of type 1 diabetes in childhood has been reported from most regions worldwide [2, 3] strongly suggesting that there are modifiable risk factors involved in its aetiology. Although not conclusive, evidence suggests that the origin of this increase dates from the middle of the last century [4]. It has been noted that this increase parallels the introduction of mass childhood vaccination programmes in developed countries [5]. This has led to speculation that the immune-modifying effects of vaccinations in early childhood, particularly at a formative age for the developing immune system, might play a part in the rising incidence of type 1 diabetes. Infectious diseases could play a part in the pathogenesis of type 1 diabetes by disturbing the developing immune system and so interfering with its future self-/nonself-discrimination capacity [6]. This rationale may be related to the hypothesis that limited exposure to infections and more extensive use of vaccinations early in life can lead to an increased risk of childhood diabetes [7, 8]. A lack of exposure to infections may limit the education of T cell repertoire in the developing immune system and, as a result, there may be an inappropriate response to a subsequent infection.

Although a number of epidemiological studies have investigated routine vaccinations and type 1 diabetes, the risk estimates from individual studies often lack power because of small sample sizes. Moreover, it is difficult to make overall conclusions about vaccinations because results are spread over a large number of studies. Thus, a meta-analysis of such studies could provide a better assessment of the effect of routine vaccinations on the risk of childhood type 1 diabetes and provide a succinct summary of the literature.

Consequently, the aim of our study was to investigate the association between routine vaccinations and risk of childhood type 1 diabetes mellitus by systematically reviewing the published literature and performing meta-analysis where possible.

## Methods

### Literature search

A search strategy was developed (electronic supplementary material [ESM] Table 1) and a literature search was conducted using Ovid MEDLINE (US National Library of Medicine, Bethesda, MD, USA). A similar search strategy was adopted using EMBASE (Reed Elsevier, Amsterdam, the Netherlands). The search included articles from the earliest available date of each database (1947 for both) through to the first week of July 2013. No language restrictions were made and conference proceedings were included.

The screening process was conducted independently by two investigators (S. R. Halliday and E. Morgan). Titles from the literature search were screened and those that were obviously irrelevant were removed. Following this, titles and abstracts were reviewed to identify relevant articles. In cases where relevancy could not be determined by reviewing the abstract, full text papers were requested and assessed. Reference lists of the selected articles were manually screened to identify articles that were not found in the initial search and further searches were performed for articles that had cited any of the articles already identified.

Articles were reviewed based on the following inclusion criteria: studies assessed vaccinations in children and young adults diagnosed with type 1 diabetes; studies identified a comparable group of participants without type 1 diabetes; studies reported the prevalence of vaccination history in these two groups or reported ORs or RRs for the association between vaccination history and type 1 diabetes.

Study characteristics were independently extracted from the published reports by two investigators (G. R. Campbell and S. R. Halliday). The details recorded were author, date of publication, country, source of patients, age at diagnosis, source of controls, and definition and source of information about childhood vaccinations.

The quality of papers included in this review was assessed independently by two investigators (S. R. Halliday and E. Morgan) using the Newcastle–Ottawa scale (NOS) [9]. A threshold score of seven or more was used to identify the higher quality articles.

### Statistical analysis

Adjusted and unadjusted ORs or RRs and their SEs were extracted from each study and entered into Review Manager version 5.1 (Nordic Cochrane Centre, Copenhagen, Denmark). When estimates were provided for a combined vaccine, the same estimate was used for the analysis of each individual vaccine. To account for the anticipated heterogeneity between studies, a random effects model was used to calculate an overall pooled estimate. A *χ*^2^ test was obtained and the *I*^2^ statistic used to measure the extent of heterogeneity [10]. For each vaccination, an initial analysis was conducted using published unadjusted estimates, or age-adjusted estimates when available. Further analyses were conducted using estimates which were adjusted for additional covariates if they had been provided. Sensitivity analyses were conducted to investigate whether results differed on the basis of study design (case–control or nested case–control/cohort) or quality score (NOS score of seven or more or less than seven). To check for publication bias, funnel plots were produced by plotting the OR for each study against the SE of the natural logarithm of the OR, and Egger tests were conducted to investigate funnel plot asymmetry [11].

## Results

The results of the literature search are shown in ESM Fig. 1. The MEDLINE and EMBASE searches yielded 2,156 articles. After screening titles and abstracts, 2,113 articles were excluded, leaving 43 full text articles to be screened for eligibility. Following this screen, 33 were excluded (listed in ESM Table 2), leaving ten eligible articles [5, 6, 12–19]. Two additional articles were identified by searching the reference lists of these ten articles [20, 21]. Finally, five more articles were identified as having cited one or more of the already identified articles [22–26]. One of the identified articles had conducted two separate case–control studies, one using controls from a community-based survey and the other using friends and neighbourhood controls matched by age and sex [13]. The multicentre EURODIAB publication [6] reported estimates from seven separate centres with individual patient data available, but one of these was omitted because the results had been previously presented in a separate publication [15]. In total, 13,323 cases of childhood diabetes from 23 studies were included in the 17 articles that contributed to this analysis.

Characteristics of the included studies are shown in Table 1. They were predominantly case–control in design including two nested case–control studies, with an additional four cohort studies and one randomised controlled trial. Eleven of the articles were from Europe, two from Australia [20, 21] and the US [16, 18], and one each from Canada [13] and Africa [22]. Type 1 diabetes cases were ascertained through a diabetes register in seven articles.

**Table 1:** Characteristics of studies investigating the association between vaccinations and type 1 diabetes, ordered by publication date

First author, year^a^ [reference]	Design	NOS score	Cases			Controls			Vaccination history
			*n*	Age at dx (years)	Source (years of dx)	*n*	Age (years)	Source (matching criteria)	
Glatthaar (1988) [21]	C-C	<7	194	<15	Western Australia school survey (1972–1984)	753	<15	Western Australia school class lists (age, sex)	Questionnaire
Blom (1991) [12]	C-C	≥7	339	<15	Swedish national diabetes register (1985–1986)	538	<15	Swedish population register (age, sex, county)	Questionnaire, child/school health record
Telahun (1994) [22]	C-C	<7	41	<15	Two Ethiopian hospital diabetes clinics (prior to 1992)	99	<15	Admissions to one Ethiopian hospital	Questionnaire, immunisation card
Verge (1994) [20]	C-C	≥7	217	<15	New South Wales diabetes register (1990–1991)	258	<15	Schools, pre-schools and day-care centres (age, sex)	Questionnaire
Parent (1997) [13]									
Series A	C-C	≥7	93	<19	Montreal diabetes register (1976–1991)	2,903	<19	Community survey (age, locality)	Central vaccination register
Series B	C-C	≥7	249	<19	Montreal diabetes register (1982–1986)	431	<19	Friends (age, sex, locality)	Central vaccination register
Karvonen (1999) [14]	Cohort	≥7	886	<11	Finnish national diabetes register & hospital discharges (1983–1997)	372,901	<11	Finnish medical birth registry	Vaccination trial
Rami (1999) [15]	C-C	≥7	114	<15	Vienna diabetes register (1989–1994)	495	<15	Vienna Schools Registers (age, sex)	Questionnaire, vaccination file
EURODIAB (2000) [6]									
Latvia	C-C	≥7	141	<15	Latvia national diabetes register (1989–1994)	324	<15	Population-based register (age)	Interview, medical record
Lithuania	C-C	≥7	117	<15	Lithuania national diabetes register (1989–1994)	269	<15	Policlinics (age)	Questionnaire, medical record
Luxemburg	C-C	≥7	59	<15	Luxembourg national diabetes register (1989–1995)	178	<15	Schools/pre-schools (age)	Interview, medical record
Bucharest	C-C	≥7	82	<15	Bucharest diabetes register (1989–1994)	277	<15	Health service register (age)	Interview, medical record
Yorkshire	C-C	≥7	208	<15	Yorkshire diabetes register (1993–1994)	409	<15	General practitioner registers (age)	Interview, medical record
Northern Ireland	C-C	≥7	189	<15	Northern Ireland diabetes register (1990–1992)	465	<15	General practitioner registers (age)	Questionnaire, medical record
DeStefano (2001) [16]	NC-C	≥7	252	<11	US health maintenance organisation records (1988–1997)	768	<11	Enrolment files (sex, age, length of enrolment)	Medical record
Black (2002) [18]	RCT	<7	8,605	<13	Clinical records in the Northern California Kaiser Permanente Medical Care Program	35,373	<13	Programme participants	Treatment allocation
Montgomery (2002) [17]	Cohort	≥7	13	<10	British Cohort Study (1970–1980)	7,079	<10	British Cohort Study (age)	Interview
Altobelli (2003) [23]	C-C	<7	136	<15	Abruzzo diabetes register (1990–1996)	272	<15	National Health System (age)	Questionnaire, National Health System
Hviid (2004) [5]	Cohort	≥7	681	<12	Danish National Hospital Register (1990–2001)	739,013	<12	Danish Civil Registration System	National Board of Health Register
Cardwell (2008) [24]	NC-C	≥7	367	<15	UK General Practice Research Database	4,579	<15	General Practice Research Database (age, sex, region)	Medical record
Karavanaki (2008) [19]	C-C	<7	127	<16	Two paediatric hospitals in Athens (1999–2000)	150	<15	Hospital admissions	Questionnaire
Skrodenienė (2010) [25]	C-C	<7	124	<15	Kaunas University Clinic, Lithuania (1996–2000)	78	<15	Outpatient department (age, sex)	Questionnaire
Bardage (2011) [26]	Cohort	≥7	89	<20	Healthcare register for Stockholm County Council (2009–2010)	468,927	<20	Population-based register	Vaccination register

C-C, case–control; dx, diagnosis; NC-C, nested case–control; RCT, randomised control trial

^a^Year of publication

All of the cohort studies accounted for age in their analyses and all but two of the case–control studies matched controls to cases by age [19, 22]. Most studies also matched controls by sex. Further matching variables included location [12, 13, 24] and length of enrolment [16]. Four articles provided estimates after further adjustment for maternal educational level [20], race/ethnicity and family history of type 1 diabetes [16], calendar period [5], and healthcare consumption [26].

Methods of exposure ascertainment varied across studies (Table 1). Ten of the 23 studies used a combination of interview/questionnaires and medical records, seven studies solely used medical records or register data, and the remaining five studies obtained exposure data purely from recall [17, 19–21, 25]. Participants in one article were divided into three birth cohorts to investigate the timing of vaccinations; cohort 1 consisted of control participants who had not been vaccinated at all and cohorts 2 and 3 had been vaccinated at different ages and calendar periods [14]. Published response rates varied from 55% to 100%, although some studies did not report response rates. However, this forms part of the NOS assessment.

Studies investigated a wide range of vaccinations (ESM Table 3), of which measles, rubella, mumps and pertussis were the most commonly investigated, while H1N1 influenza, tick-borne encephalitis, hepatitis B, meningitis C and smallpox were each reported in only one of the 17 articles.

### Quality assessment

The quality of each study report was assessed by two independent reviewers (E. Morgan and S. R. Halliday) using the NOS. Eleven of the 17 reports [5, 6, 12–17, 20, 24, 26] were rated as being of “good” quality, with an NOS score of seven or more.

### Overall findings

Results from the meta-analyses are summarised in Table 2. Both minimally adjusted and further adjusted pooled estimates are presented. The upper line of data shows the pooled results for all studies while the lower line shows the pooled results after the elimination of any results from the six studies which scored less than seven on the NOS. Forest plots of the minimally adjusted estimates for each type of vaccination are presented in ESM Fig. 2.

**Table 2:** Summary of meta-analyses results for vaccinations as risk factors for childhood type 1 diabetes

Vaccination^a^	Studies (*n*)	Cases (*n*)	Controls (*n*)	Unadjusted/minimally adjusted analysis				Fully adjusted analysis			
				OR (95% CI)	*p*	*I* ^2^ (%)	Hetero *p*	OR (95% CI)	*p*	*I* ^2^ (%)	Hetero *p*
Morbilli/measles											
All	15	2,822	9,314^b^	0.75 (0.54, 1.05)	0.09	84	<0.001	0.75 (0.54, 1.05)	0.09	84	<0.001
NOS ≥7	11	2,324	8,040^b^	1.03 (0.88, 1.21)	0.70	15	0.3	1.03 (0.88, 1.21)	0.71	15	0.3
Rubella/German measles											
All	14	2,776	9,221^b^	0.85 (0.58, 1.26)	0.42	84	<0.001	0.85 (0.58, 1.26)	0.43	84	<0.001
NOS ≥7	11	2,319	8,046^b^	1.15 (0.96, 1.37)	0.14	21	0.25	1.15 (0.96, 1.37)	0.14	20	0.25
Parotitis/mumps											
All	13	2,690	8,409^b^	0.81 (0.60, 1.12)	0.20	84	<0.001	0.81 (0.59, 1.11)	0.20	84	<0.001
NOS ≥7	11	2,427	7,987^b^	1.03 (0.90, 1.17)	0.67	5	0.4	1.03 (0.90, 1.17)	0.70	9	0.36
Pertussis/whooping cough											
All	14	1,896	15,720	0.79 (0.53, 1.19)	0.26	74	<0.001	0.79 (0.53, 1.18)	0.25	74	<0.001
NOS ≥7	10	1,401	14,518	0.92 (0.71, 1.20)	0.55	27	0.2	0.92 (0.70, 1.21)	0.51	36	0.13
BCG											
All	11	1,869	10,685	0.96 (0.76, 1.21)	0.75	25	0.2	0.96 (0.76, 1.21)	0.73	26	0.2
NOS ≥7	10	1,828	10,586	1.02 (0.85, 1.22)	0.87	0	0.53	1.01 (0.84, 1.22)	0.88	0	0.51
HiB											
All	11	11,828	415,048^b^	1.04 (0.94, 1.14)	0.46	4	0.40	1.07 (0.97, 1.17)	0.17	0	0.61
NOS ≥7	10	3,223	379,675^b^	1.06 (0.96, 1.16)	0.24	0	0.47	1.08 (0.98, 1.19)	0.10	0	0.73
Tetanus											
All	10	1,361	3,330	0.76 (0.43, 1.34)	0.34	63	0.004	0.76 (0.43, 1.34)	0.34	63	0.004
NOS ≥7	7	1,002	2,400	0.80 (0.47, 1.34)	0.39	40	0.13	0.80 (0.47, 1.34)	0.39	40	0.13
Diphtheria											
All	9	1,015	2,686	0.99 (0.49, 2.00)	0.98	61	0.008	0.99 (0.49, 2.00)	0.98	61	0.008
NOS ≥7	6	656	1,756	1.02 (0.56, 1.85)	0.95	19	0.29	1.02 (0.56, 1.85)	0.95	19	0.29
Poliomyelitis											
All	8	1,040	2,481	0.80 (0.39, 1.68)	0.56	54	0.03	0.80 (0.39, 1.68)	0.56	54	0.03
NOS ≥7	7	999	2,382	1.07 (0.61, 1.88)	0.82	5	0.39	1.07 (0.61, 1.88)	0.82	5	0.39
MMR											
All	5	1,505	5,469^b^	0.58 (0.24, 1.40)	0.22	92	<0.001	0.53 (0.21, 1.38)	0.19	94	<0.001
NOS ≥7	3	1,242	5,347^b^	1.21 (0.99, 1.49)	0.07	0	0.56	1.21 (0.98, 1.49)	0.07	0	0.57
DTP											
All	3	359	930	0.90 (0.15, 5.31)	0.91	84	0.002	0.90 (0.15, 5.31)	0.91	84	0.002
NOS ≥7	0	0	0	NA	NA	NA	NA	NA	NA	NA	NA

Hetero *p*, *p* value for heterogeneity; NA, not applicable

^a^NOS ≥7 – analyses restricted to high quality studies which have an NOS score of seven or more

^b^Excludes one study with an unstated number of controls

Meta-analyses found no significant association between any of the 11 vaccinations and the risk of type 1 diabetes. For instance, a meta-analysis of the 15 studies that investigated measles found a non-significant decrease in the risk of developing type 1 diabetes (OR 0.75 [95% CI 0.54, 1.05]; *p* = 0.09), with strong evidence of heterogeneity (*I*^2^ = 84%, *p* for heterogeneity <0.001). Results were minimally changed after adjusted estimates were used. There was a high degree of heterogeneity observed in most of the meta-analyses except for studies investigating haemophilus influenza B (HiB) and Bacillus Calmette–Guérin (BCG) vaccinations. None of the Egger tests for the 11 vaccinations showed significant evidence of funnel plot asymmetry, which would be indicative of publication bias.

Results of the remaining vaccinations that were reported in only one study each showed no significant associations with H1N1 influenza, tick-borne encephalitis, hepatitis B, meningitis C or smallpox.

### Sensitivity analyses

Further investigations of the associations between vaccinations and risk of type 1 diabetes were conducted in a number of sensitivity analyses.

Removal of the six studies that scored less than seven on the NOS resulted in the levels of heterogeneity being considerably reduced (Table 2, lower data lines). The pooled ORs were little affected by this exclusion and all remained statistically non-significant.

Another sensitivity analysis was conducted to assess whether there was a difference in the overall effect due to study design; however, the estimates remained largely unchanged (results not shown).

## Discussion

This study found no evidence that any of the reported vaccinations were associated with the risk of childhood type 1 diabetes. These findings were little altered after adjustment for potentially confounding factors. Results were also largely unchanged after two sensitivity analyses investigating the effect of study design and quality assessment score were conducted.

The rationale that immunisations contribute to the risk of type 1 diabetes is not supported by this systematic review. Our findings in humans do not support animal studies which have suggested that vaccination timing affects diabetes development in rodents [27]. Although studies have found that countries without vaccination programmes have lower rates of type 1 diabetes and that diabetes rates have increased over time with the introduction of vaccination programmes [28], our findings suggest that these differences are not due to the vaccination programmes but rather to other differences between countries or to other changes over time. In support of our findings, the Diabetes Autoimmunity Study found that there was no increase in the risk of developing beta-cell autoimmunity or type 1 diabetes with vaccination (including hepatitis B, HiB, polio or diphtheria, tetanus and pertussis [DTP]) or the timing of vaccinations [29]. However, due to the small number of cases of beta cell autoimmunity or type 1 diabetes (*n* = 25), this study lacked sufficient statistical power to detect an association, and results should be interpreted with caution.

As far as we are aware, this is the first systematic review and meta-analyses to investigate the association of routine vaccinations and risk of childhood type 1 diabetes. This systematic review benefits from extensive searches and the inclusion of data on 13,323 children with type 1 diabetes, thus providing a sufficiently high statistical power to detect even modest associations and having narrow 95% CIs for the OR estimates. Most studies used clinical records or registers to ascertain vaccination status and of those that were questionnaire- or interview-based, most used medical records to validate vaccination data and eliminate the risk of potential recall bias.

Notwithstanding these strengths, there were a number of limitations for this systematic review. Too few studies stratified data according to the timing of vaccinations to adequately assess whether the associations differed by timing of vaccination as postulated by Classen and Classen [27, 28]. Only two articles gave further detail on the timing of vaccinations [14, 16]. In the Finnish study, Karvonen et al reported no difference in the risk of type 1 diabetes in a cohort vaccinated against HiB at age 3 months compared with a cohort vaccinated at 24 months of age [14]. Similarly, a later US study did not find any association with diabetes risk when infants were vaccinated against hepatitis B at birth compared with those who received their first vaccination later in life [16].

Although the marked heterogeneity in the estimates for most vaccinations is a further weakness of our study, this was largely reduced when analyses were restricted to higher quality studies.

This systematic review was limited in the variation of confounders adjusted for among studies, which could have resulted in residual confounding. The predominance of retrospective case–control studies in this review should be recognised as a further limitation because the possibility remains that participation was different for parents of case and control participants and that participation rates may be higher in health conscious parents who may be more likely to have their children vaccinated. The reliance on parental recall of vaccinations in five studies [17, 19–21, 25] can have limitations, particularly for differences in recall between parents of case and control children [30]. It could be that the parents of children with type 1 diabetes are more mindful of their children’s vaccination history. Alternatively, the strain and worry of having a child with type 1 diabetes may overshadow the memory of what vaccinations they had received at birth and consequently lead to under-reporting. More recently established prospective birth cohort studies should provide reliable vaccination information [31]. However, even though cohort participants are selected as being at a high risk, the small numbers of cases of diabetes together with the uniformly high rates of vaccination attained in many countries may mean that their power to detect associations is limited.

In conclusion, this systematic review and meta-analysis does not show evidence of an association between any of the routine vaccinations investigated and childhood type 1 diabetes. Therefore, this study does not provide a rationale for altering vaccination programmes based upon a subsequent risk of type 1 diabetes. Future research in this area should focus on the timing of vaccinations because mouse models suggest this may be important, and only two studies included in this review investigated vaccination timing [14, 16].

## Electronic supplementary material

ESM Fig. 1 (PDF 22.6 kb)

ESM Fig. 2 (PDF 227 kb)

ESM Table 1 (PDF 13.9 kb)

ESM Table 2 (PDF 172 kb)

ESM Table 3 (PDF 132 kb)

## Abbreviations

BCG: Bacillus Calmette–Guérin
DTP: Diphtheria, tetanus and pertussis
HiB: Haemophilus influenza B
MMR: Measles, mumps and rubella
NOS: Newcastle–Ottawa scale
