# Supplementary figures and images for: Vaccinations and childhood type 1 diabetes mellitus: a meta-analysis of observational studies
Source: Diabetologia. 2015 Nov 12;59:237–43. doi: 10.1007/s00125-015-3800-8 (PMC4705121; doi:10.1007/s00125-015-3800-8)

**ESM Figure 1:** Flow chart of search results

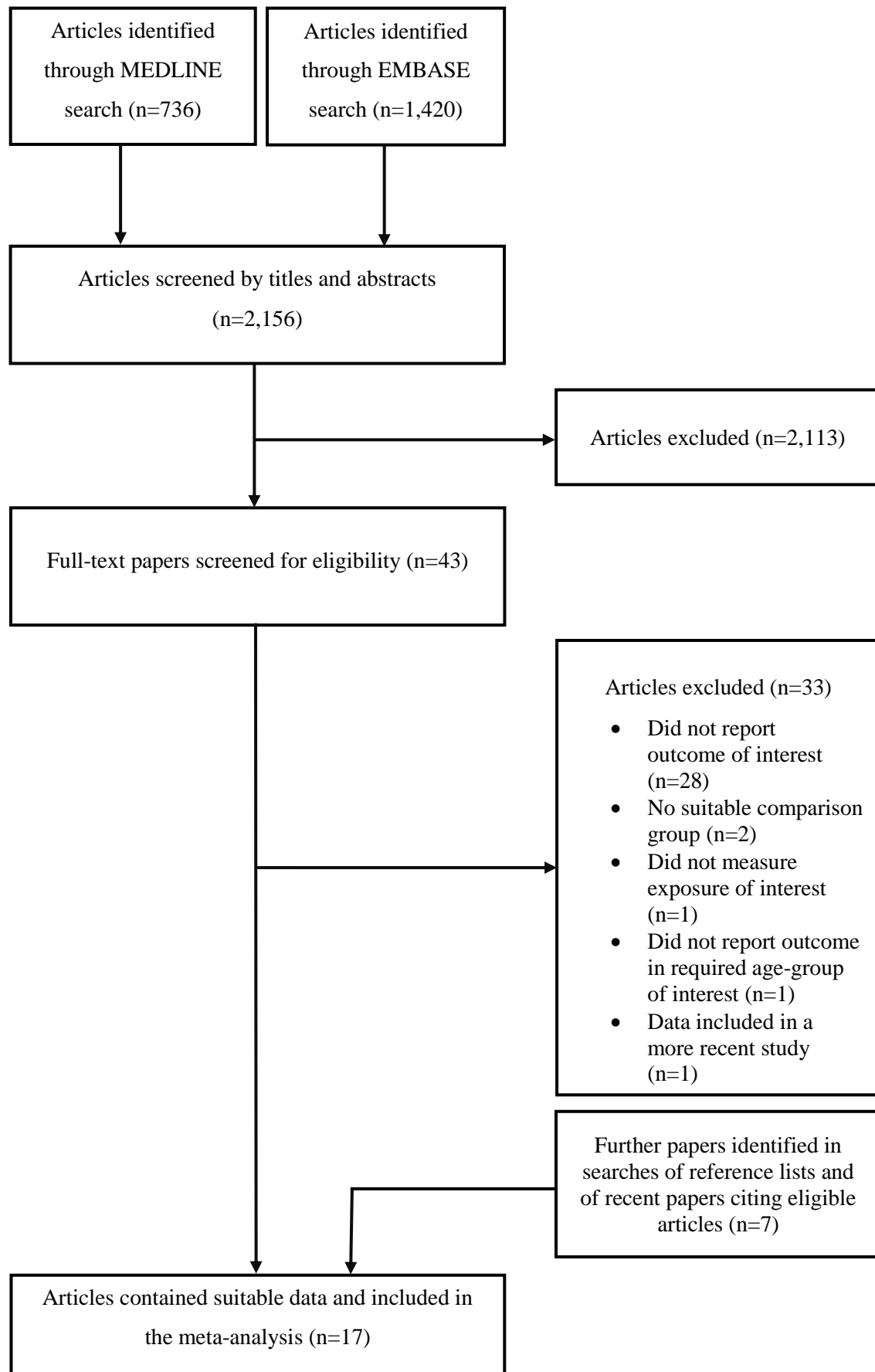

Supplement: Supplementary file 1 — (PDF 22.6 kb) [file 125_2015_3800_MOESM1_ESM.pdf]
