# Supplementary material for: Vaccinations and childhood type 1 diabetes mellitus: a meta-analysis of observational studies
Source: Diabetologia. 2015 Nov 12;59:237–43. doi: 10.1007/s00125-015-3800-8 (PMC4705121; doi:10.1007/s00125-015-3800-8)
Supplement: Supplementary file 2 — (PDF 227 kb) [file 125_2015_3800_MOESM2_ESM.pdf]

Measles

Study or Subgroup	Cases Total	Controls Total	Weight	Odds Ratio IV, Random, 95% CI
Glatthaar et al (1988) [21]	194	753	8.0%	1.22 [0.86, 1.74]
Blom et al (1991) [12]	348	645	7.9%	0.69 [0.48, 0.98]
Telahun et al (1994) [22]	41	99	5.8%	0.26 [0.12, 0.57]
Rami et al (1999) [15]	114	495	7.4%	0.95 [0.59, 1.54]
EURODIAB - Latvia (2000) [6]	129	283	6.5%	0.81 [0.42, 1.54]
EURODIAB - Lithuania (2000) [6]	116	268	6.4%	1.11 [0.56, 2.20]
EURODIAB - Luxembourg (2000) [6]	52	172	6.3%	1.05 [0.52, 2.11]
EURODIAB - Romania (2000) [6]	82	277	3.1%	0.39 [0.08, 1.76]
EURODIAB - UK Yorkshire (2000) [6]	59	101	6.0%	1.50 [0.71, 3.16]
EURODIAB - UK NI (2000) [6]	182	452	8.0%	1.04 [0.74, 1.47]
DeStefano et al (2001) [16]	252	768	6.5%	1.36 [0.70, 2.63]
Altobelli et al (2003) [23]	136	272	7.6%	0.31 [0.20, 0.48]
Hviid et al (2004) [5]	623	?	8.4%	1.14 [0.90, 1.45]
Cardwell et al (2008) [24]	367	4579	7.1%	1.56 [0.90, 2.69]
Karavanaki et al (2008) [19]	127	150	5.2%	0.05 [0.02, 0.13]
**Total (95% CI)**	**2822**	**9314**	**100.0%**	**0.75 [0.54, 1.05]**

Heterogeneity: Tau² = 0.32; Chi² = 88.76, df = 14 (P < 0.00001); I² = 84%
Test for overall effect: Z = 1.69 (P = 0.09)

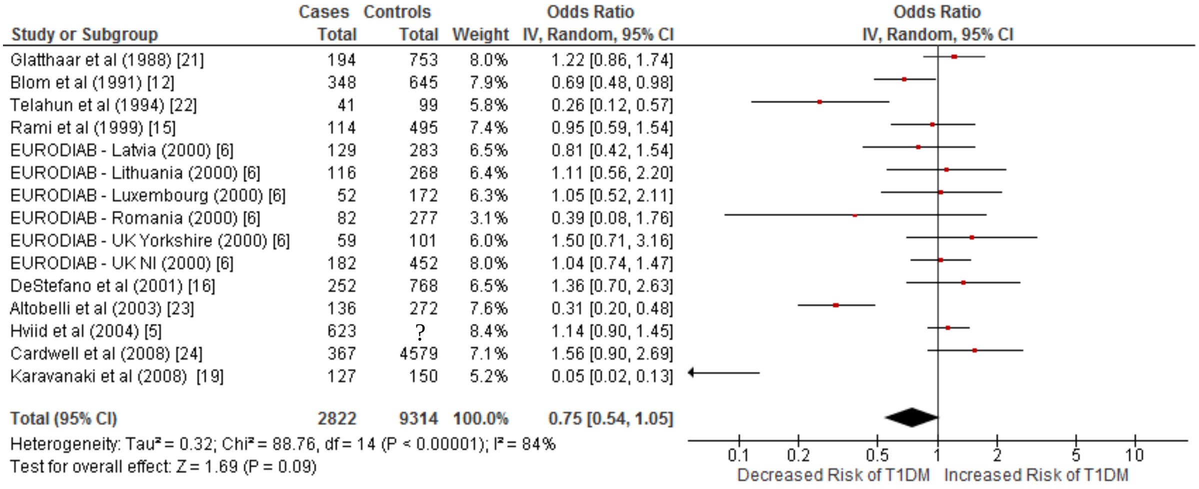

Rubella

Study or Subgroup	Cases Total	Controls Total	Weight	Odds Ratio IV, Random, 95% CI
Glatthaar et al (1988) [21]	194	753	7.4%	1.04 [0.47, 2.29]
Blom et al (1991) [12]	348	645	10.0%	0.95 [0.71, 1.28]
Rami et al (1999) [15]	114	495	7.8%	0.50 [0.24, 1.02]
EURODIAB - Latvia (2000) [6]	136	308	2.1%	1.15 [0.10, 12.74]
EURODIAB - Lithuania (2000) [6]	117	268	3.3%	3.50 [0.58, 21.23]
EURODIAB - Luxembourg (2000) [6]	52	174	8.2%	1.75 [0.91, 3.36]
EURODIAB - Romania (2000) [6]	82	277	1.4%	0.67 [0.03, 14.05]
EURODIAB - UK Yorkshire (2000) [6]	44	77	7.4%	1.79 [0.81, 3.94]
EURODIAB - UK NI (2000) [6]	184	455	9.8%	1.08 [0.77, 1.53]
DeStefano et al (2001) [16]	252	768	8.1%	1.36 [0.70, 2.63]
Altobelli et al (2003) [23]	136	272	9.0%	0.28 [0.17, 0.46]
Hviid et al (2004) [5]	623	?	10.2%	1.14 [0.90, 1.45]
Cardwell et al (2008) [24]	367	4579	8.8%	1.56 [0.90, 2.69]
Karavanaki et al (2008) [19]	127	150	6.7%	0.05 [0.02, 0.13]
**Total (95% CI)**	**2776**	**9221**	**100.0%**	**0.85 [0.58, 1.26]**

Heterogeneity: Tau² = 0.38; Chi² = 80.62, df = 13 (P < 0.00001); I² = 84%
Test for overall effect: Z = 0.80 (P = 0.42)

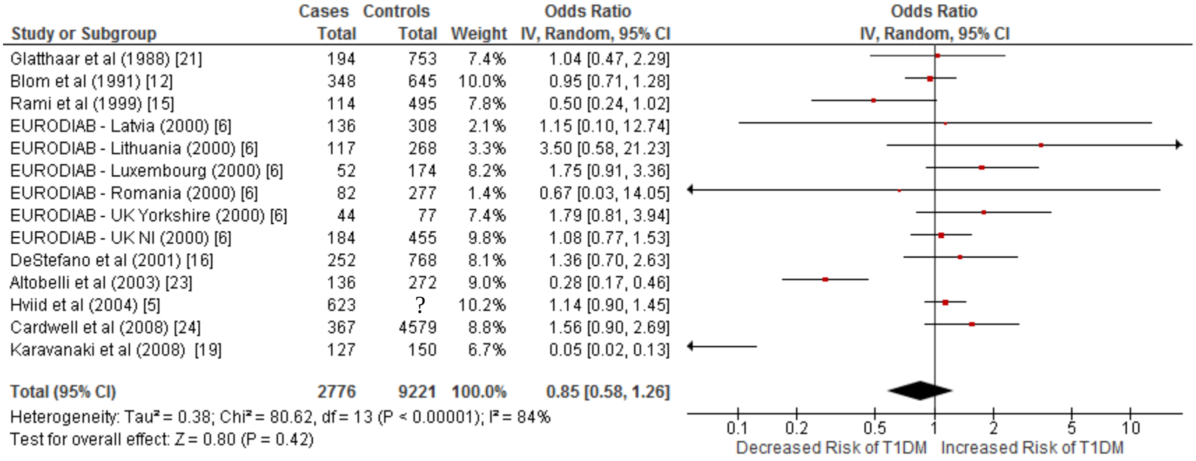

Mumps

Study or Subgroup	Cases Total	Controls Total	Weight	Odds Ratio IV, Random, 95% CI
Blom et al (1991) [12]	348	645	9.1%	0.95 [0.70, 1.27]
Verge et al (1994) [20]	198	241	8.1%	0.70 [0.44, 1.11]
Rami et al (1999) [15]	114	495	8.1%	1.05 [0.65, 1.68]
EURODIAB - Latvia (2000) [6]	130	285	8.3%	0.80 [0.52, 1.24]
EURODIAB - Lithuania (2000) [6]	117	269	8.2%	0.84 [0.54, 1.32]
EURODIAB - Luxembourg (2000) [6]	52	174	6.8%	1.27 [0.64, 2.49]
EURODIAB - UK Yorkshire (2000) [6]	42	73	5.8%	1.75 [0.76, 4.04]
EURODIAB - UK NI (2000) [6]	184	458	8.7%	1.02 [0.71, 1.46]
DeStefano et al (2001) [16]	252	768	6.9%	1.36 [0.70, 2.63]
Altobelli et al (2003) [23]	136	272	7.9%	0.28 [0.17, 0.47]
Hviid et al (2004) [5]	623	?	9.3%	1.14 [0.90, 1.45]
Cardwell et al (2008) [24]	367	4579	7.6%	1.56 [0.90, 2.69]
Karavanaki et al (2008) [19]	127	150	5.4%	0.05 [0.02, 0.13]
**Total (95% CI)**	**2690**	**8409**	**100.0%**	**0.81 [0.60, 1.12]**

Heterogeneity: Tau² = 0.26; Chi² = 73.50, df = 12 (P < 0.00001); I² = 84%
Test for overall effect: Z = 1.28 (P = 0.20)

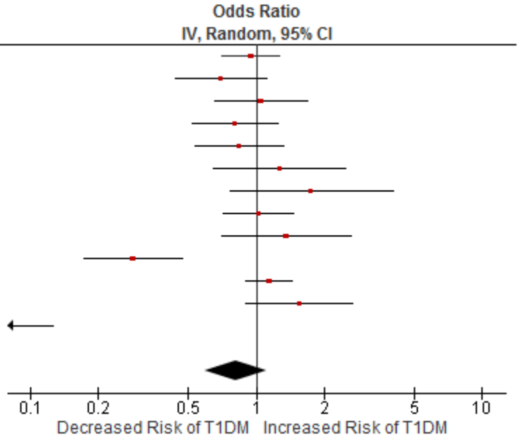

ESM Figure 2: Forest plot of vaccinations and type 1 diabetes using the random effects model based on inverse variance (IV) weighting, studies ordered by publication date using age-adjusted estimates where available. Chi^2, χ^2; T1DM, Type 1 diabetes mellitus.

Pertussis

Study or Subgroup	Cases Total	Controls Total	Weight	Odds Ratio IV, Random, 95% CI
Glatthaar et al (1988) [21]	194	753	4.2%	0.94 [0.19, 4.52]
Telahun et al (1994) [22]	41	99	7.8%	0.24 [0.11, 0.54]
Rami et al (1999) [15]	114	495	10.1%	0.64 [0.42, 0.98]
EURODIAB - Latvia (2000) [6]	129	284	7.5%	0.74 [0.32, 1.75]
EURODIAB - Lithuania (2000) [6]	116	266	8.2%	1.61 [0.77, 3.36]
EURODIAB - Luxembourg (2000) [6]	54	173	5.4%	1.85 [0.52, 6.58]
EURODIAB - Romania (2000) [6]	82	277	1.5%	1.50 [0.07, 31.50]
EURODIAB - UK Yorkshire (2000) [6]	97	167	9.6%	1.16 [0.70, 1.95]
EURODIAB - UK NI (2000) [6]	177	430	10.4%	0.77 [0.53, 1.12]
DeStefano et al (2001) [16]	252	768	5.1%	0.28 [0.07, 1.06]
Montgomery et al (2002) [17]	13	7079	8.3%	1.15 [0.56, 2.37]
Altobelli et al (2003) [23]	136	272	9.5%	0.21 [0.12, 0.35]
Cardwell et al (2008) [24]	367	4579	7.7%	1.34 [0.59, 3.08]
Skrodeniene et al (2010) [25]	124	78	4.8%	4.02 [0.99, 16.32]
**Total (95% CI)**	**1896**	**15720**	**100.0%**	**0.79 [0.53, 1.19]**

Heterogeneity: Tau² = 0.37; Chi² = 50.02, df = 13 (P < 0.00001); I² = 74%
Test for overall effect: Z = 1.12 (P = 0.26)

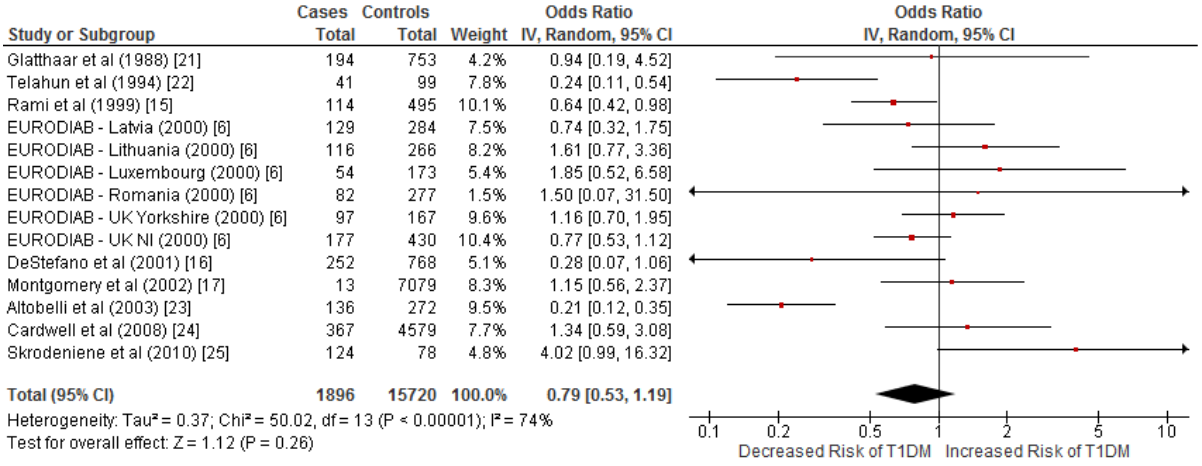

BCG

Study or Subgroup	Cases Total	Controls Total	Weight	Odds Ratio IV, Random, 95% CI
Blom et al (1991) [12]	348	645	24.1%	1.04 [0.77, 1.40]
Telahun et al (1994) [22]	41	99	6.4%	0.37 [0.16, 0.86]
Parent et al (1997) [13] - Series A	93	2903	11.9%	1.09 [0.62, 1.92]
Parent, 1997 Series B	249	431	15.3%	1.26 [0.79, 2.01]
Rami et al (1999) [15]	114	495	3.8%	0.80 [0.26, 2.48]
EURODIAB - Latvia (2000) [6]	134	300	0.9%	0.89 [0.08, 9.93]
EURODIAB - Lithuania (2000) [6]	117	269	3.0%	1.62 [0.44, 5.92]
EURODIAB - Luxembourg (2000) [6]	52	175	7.9%	1.66 [0.79, 3.48]
EURODIAB - UK Yorkshire (2000) [6]	171	342	2.8%	1.61 [0.43, 6.09]
EURODIAB - UK NI (2000) [6]	183	447	16.3%	0.65 [0.42, 1.01]
Cardwell et al (2008) [24]	367	4579	7.4%	0.80 [0.37, 1.73]
**Total (95% CI)**	**1869**	**10685**	**100.0%**	**0.96 [0.76, 1.21]**

Heterogeneity: Tau² = 0.04; Chi² = 13.36, df = 10 (P = 0.20); I² = 25%
Test for overall effect: Z = 0.32 (P = 0.75)

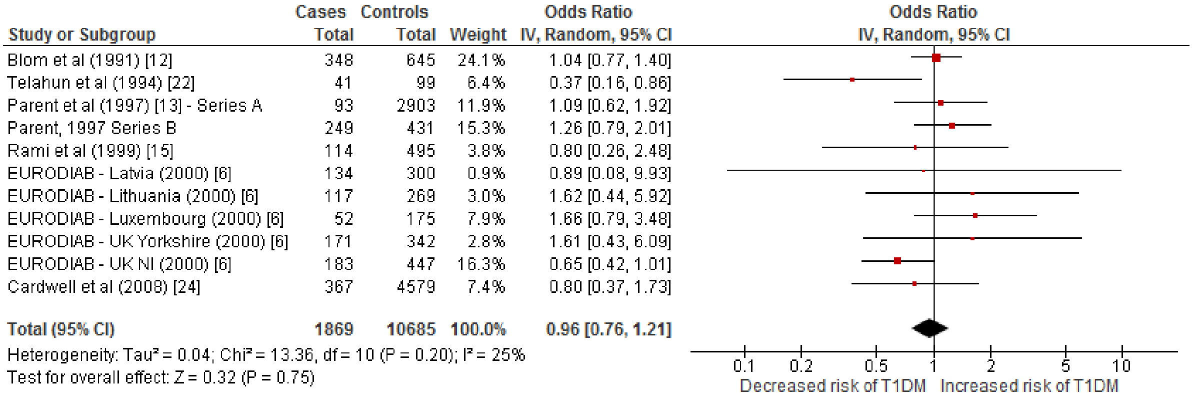

HiB

Study or Subgroup	Cases Total	Controls Total	Weight	Odds Ratio IV, Random, 95% CI
Black et al (2002) [18]	8605	35373	4.9%	0.78 [0.51, 1.20]
Cardwell et al (2008) [24]	367	4579	10.8%	0.84 [0.63, 1.13]
DeStefano et al (2001) [16]	252	468	1.4%	1.14 [0.51, 2.57]
EURODIAB - Luxembourg (2000) [6]	54	172	0.8%	1.15 [0.39, 3.36]
EURODIAB - Romania (2000) [6]	82	276	0.1%	0.47 [0.02, 9.26]
EURODIAB - UK NI (2000) [6]	179	443	0.1%	7.45 [0.30, 183.83]
EURODIAB - UK Yorkshire (2000) [6]	171	341	1.3%	1.30 [0.55, 3.06]
Hviid et al (2004) [5]	681	?	19.6%	0.91 [0.74, 1.12]
Karvonen et al (1999) [14] - Cohort 2 (vs 1)	672	187502	31.2%	1.17 [1.00, 1.37]
Karvonen et al (1999) [14] - Cohort 3 (vs 1)	651	185399	29.6%	1.11 [0.94, 1.30]
Rami et al (1999) [15]	114	495	0.2%	0.54 [0.07, 4.35]
**Total (95% CI)**	**11828**	**415048**	**100.0%**	**1.04 [0.94, 1.14]**

Heterogeneity: Tau² = 0.00; Chi² = 10.45, df = 10 (P = 0.40); I² = 4%
Test for overall effect: Z = 0.73 (P = 0.46)

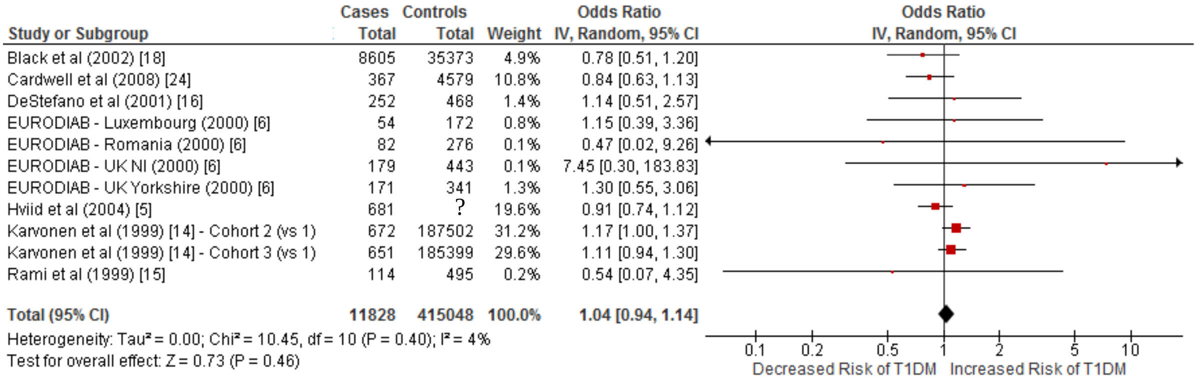

ESM Figure 2 *continued:* Forest plot of vaccinations and type 1 diabetes using the random effects model based on inverse variance (IV) weighting, studies ordered by publication date using age-adjusted estimates where available. Chi^2, χ^2; T1DM, Type 1 diabetes mellitus.

Poliomyelitis

Study or Subgroup	Cases Total	Controls Total	Weight	Odds Ratio IV, Random, 95% CI
Blom et al (1991) [12]	348	645	10.1%	1.04 [0.17, 6.25]
Telahun et al (1994) [22]	41	99	19.6%	0.24 [0.11, 0.54]
Rami et al (1999) [15]	114	495	16.5%	1.28 [0.43, 3.79]
EURODIAB - Latvia (2000) [6]	129	283	14.0%	1.38 [0.37, 5.18]
EURODIAB - Lithuania (2000) [6]	111	249	16.1%	1.78 [0.58, 5.46]
EURODIAB - Luxembourg (2000) [6]	55	175	4.3%	0.95 [0.04, 23.76]
EURODIAB - UK Yorkshire (2000) [6]	69	119	4.9%	4.18 [0.21, 82.05]
EURODIAB - UK NI (2000) [6]	173	416	14.5%	0.27 [0.08, 0.97]
**Total (95% CI)**	**1040**	**2481**	**100.0%**	**0.80 [0.39, 1.68]**

Heterogeneity: Tau² = 0.55; Chi² = 15.34, df = 7 (P = 0.03); I² = 54%
Test for overall effect: Z = 0.58 (P = 0.56)

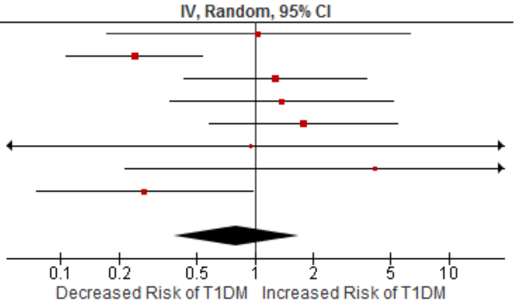

Tetanus

Study or Subgroup	Cases Total	Controls Total	Weight	Odds Ratio IV, Random, 95% CI
Glatthaar et al (1988) [21]	194	753	7.8%	0.94 [0.19, 4.52]
Blom et al (1991) [12]	348	645	18.9%	0.96 [0.70, 1.31]
Telahun et al (1994) [22]	41	99	14.2%	0.24 [0.11, 0.54]
Rami et al (1999) [15]	114	495	13.7%	0.85 [0.36, 2.00]
EURODIAB - Latvia (2000) [6]	129	283	7.3%	0.15 [0.03, 0.79]
EURODIAB - Lithuania (2000) [6]	115	266	12.2%	1.79 [0.65, 4.89]
EURODIAB - Luxembourg (2000) [6]	54	175	2.7%	0.94 [0.04, 23.33]
EURODIAB - UK Yorkshire (2000) [6]	69	119	2.9%	2.96 [0.14, 62.50]
EURODIAB - UK NI (2000) [6]	173	417	11.3%	0.35 [0.11, 1.05]
Skrodeniene et al (2010) [25]	124	78	9.0%	4.02 [0.99, 16.32]
**Total (95% CI)**	**1361**	**3330**	**100.0%**	**0.76 [0.43, 1.34]**

Heterogeneity: Tau² = 0.41; Chi² = 24.24, df = 9 (P = 0.004); I² = 63%
Test for overall effect: Z = 0.95 (P = 0.34)

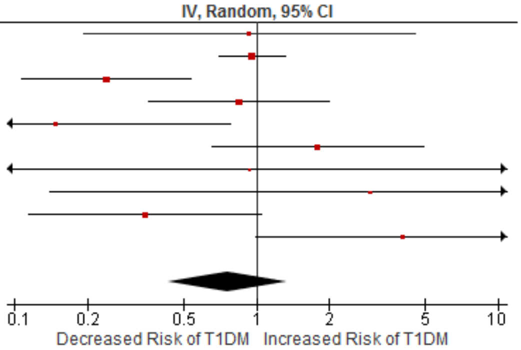

Diptheria

Study or Subgroup	Cases Total	Controls Total	Weight	Odds Ratio IV, Random, 95% CI
Glatthaar et al (1988) [21]	194	753	10.2%	0.94 [0.19, 4.52]
Telahun et al (1994) [22]	41	99	16.6%	0.24 [0.11, 0.54]
Rami et al (1999) [15]	114	495	17.2%	0.86 [0.42, 1.79]
EURODIAB - Latvia (2000) [6]	129	283	7.4%	3.72 [0.46, 30.09]
EURODIAB - Lithuania (2000) [6]	115	266	14.8%	1.79 [0.65, 4.89]
EURODIAB - Luxembourg (2000) [6]	55	174	3.9%	0.96 [0.04, 23.90]
EURODIAB - UK Yorkshire (2000) [6]	69	119	4.2%	2.96 [0.14, 62.50]
EURODIAB - UK NI (2000) [6]	174	419	14.2%	0.41 [0.14, 1.17]
Skrodeniene et al (2010) [25]	124	78	11.5%	4.02 [0.99, 16.32]
**Total (95% CI)**	**1015**	**2686**	**100.0%**	**0.99 [0.49, 2.00]**

Heterogeneity: Tau² = 0.60; Chi² = 20.57, df = 8 (P = 0.008); I² = 61%
Test for overall effect: Z = 0.03 (P = 0.98)

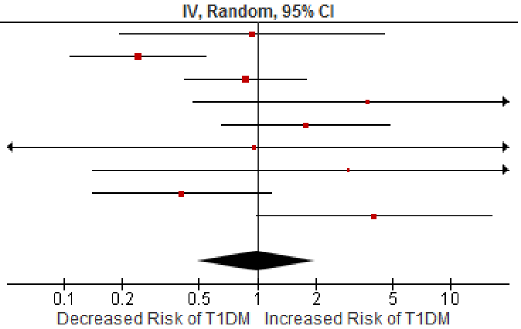

MMR

Study or Subgroup	Cases Total	Controls Total	Weight	Odds Ratio IV, Random, 95% CI
DeStefano et al (2001) [16]	252	768	19.8%	1.36 [0.70, 2.63]
Altobelli et al (2003) [23]	136	272	19.6%	0.38 [0.19, 0.76]
Hviid et al (2004) [5]	623	?	22.0%	1.14 [0.90, 1.45]
Cardwell et al (2008) [24]	367	4579	20.6%	1.56 [0.90, 2.69]
Karavanaki et al (2008) [19]	127	150	18.0%	0.05 [0.02, 0.13]
**Total (95% CI)**	**1505**	**5769**	**100.0%**	**0.58 [0.24, 1.40]**

Heterogeneity: Tau² = 0.91; Chi² = 52.86, df = 4 (P < 0.00001); I² = 92%
Test for overall effect: Z = 1.22 (P = 0.22)

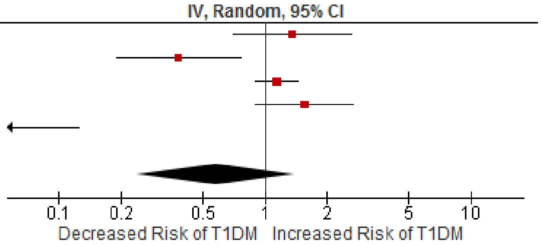

ESM Figure 2 *continued:* Forest plot of vaccinations and type 1 diabetes using the random effects model based on inverse variance (IV) weighting, studies ordered by publication date using age-adjusted estimates where available. Chi^2, χ^2; T1DM, Type 1 diabetes mellitus.

DTP

Study or Subgroup	Cases Total	Controls Total	Weight	Odds Ratio IV, Random, 95% CI
Glatthaar et al (1988) [21]	194	753	30.6%	0.94 [0.19, 4.52]
Skrodeniene et al (2010) [25]	124	78	32.2%	4.02 [0.99, 16.32]
Telahun et al (1994) [22]	41	99	37.2%	0.24 [0.11, 0.54]
**Total (95% CI)**	**359**	**930**	**100.0%**	**0.90 [0.15, 5.31]**

Heterogeneity: Tau² = 2.03; Chi² = 12.24, df = 2 (P = 0.002); I² = 84%
Test for overall effect: Z = 0.11 (P = 0.91)

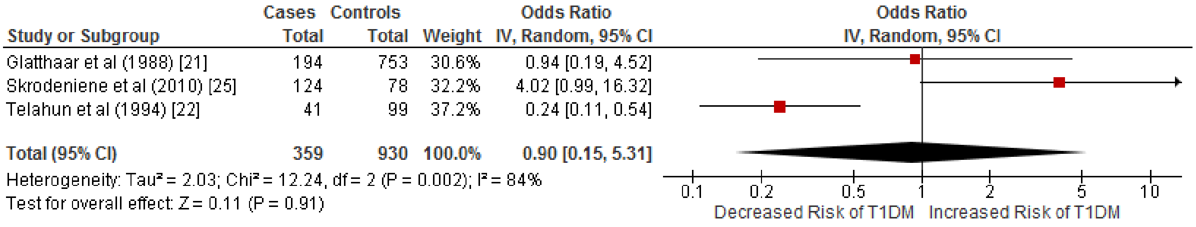

ESM Figure 2 *continued:* Forest plot of vaccinations and type 1 diabetes using the random effects model based on inverse variance (IV) weighting, studies ordered by publication date using age-adjusted estimates where available. Chi^2, χ^2; T1DM, Type 1 diabetes mellitus.
