# Supplementary material for: Vaccinations and childhood type 1 diabetes mellitus: a meta-analysis of observational studies
Source: Diabetologia. 2015 Nov 12;59:237–43. doi: 10.1007/s00125-015-3800-8 (PMC4705121; doi:10.1007/s00125-015-3800-8)
Supplement: Supplementary file 3 — (PDF 13.9 kb) [file 125_2015_3800_MOESM3_ESM.pdf]

ESM Table 1: Search strategy for used for identifying papers using MEDLINE.

**No.**	**Searches**
1	Diabetes Mellitus, Type 1/
2	IDDM.mp.
3	diabetes.mp.
4	Type 1.mp.
5	3 and 4
6	1 or 2 or 5
7	Vaccination/
8	vaccination.mp.
9	Vaccines/ or vaccine.mp.
10	measles.mp. or Measles/ or Measles Vaccine/
11	mumps.mp. or Mumps/ or Mumps Vaccine/
12	Rubella/ or Rubella Vaccine/ or rubella.mp.
13	Measles-Mumps-Rubella Vaccine/ or MMR.mp.
14	Diphtheria/ or diphtheria.mp.
15	Diphtheria-Tetanus-Pertussis Vaccine/ or Diphtheria-Tetanus-acellular Pertussis Vaccines/ or Tetanus/ or tetanus.mp. or Diphtheria-Tetanus Vaccine/
16	polio.mp. or Poliomyelitis/
17	whooping cough.mp. or Whooping Cough/
18	Haemophilus influenzae type b/ or Haemophilus/ or Haemophilus Vaccines/ or haemophilus.mp.
19	morbilli.mp.
20	parotitis.mp. or Parotitis/
21	german measles.mp.
22	pertusis.mp. or Pertussis Vaccine/
23	pneumococcal.mp. or Pneumococcal Vaccines/
24	BCG Vaccine/ or Bacillus-Calmette-Guerin.mp.
25	BCG.mp.
26	7 or 8 or 9 or 10 or 11 or 12 or 13 or 14 or 15 or 16 or 17 or 18 or 19 or 20 or 21 or 22 or 23 or 24 or 25
27	6 and 26

IDDM, insulin dependent diabetes mellitus; mp, multi-purpose.
