# Supplementary material for: Vaccinations and childhood type 1 diabetes mellitus: a meta-analysis of observational studies
Source: Diabetologia. 2015 Nov 12;59:237–43. doi: 10.1007/s00125-015-3800-8 (PMC4705121; doi:10.1007/s00125-015-3800-8)
Supplement: Supplementary file 4 — (PDF 172 kb) [file 125_2015_3800_MOESM4_ESM.pdf]

ESM Table 2: List of 33 articles excluded from review after full-text screening.

	**Excluded papers**
1	Sinaniotis CA, Daskalopoulou E, Lapatsanis P, Doxiadis S (1975) Letter: Diabetes mellitus after mumps vaccination. Arch Dis Child 50:749–750.
2	Dahlquist G, Blom L, Lönnberg G (1991) The Swedish Childhood Diabetes Study--a multivariate analysis of risk determinants for diabetes in different age groups. Diabetologia 34:757–762.
3	Dahlquist G, Gothefors L (1995) The cumulative incidence of childhood diabetes mellitus in Sweden unaffected by BCG-vaccination. Diabetologia 38:873–874.
4	Classen DC, Classen JB (1997) The timing of pediatric immunization and the risk of insulin-dependent diabetes mellitus. Infect Dis Clin Pract 6:449–454.
5	Heijbel H, Chen RT, Dahlquist G (1997) Cumulative incidence of childhood-onset IDDM is unaffected by pertussis immunization. Diabetes Care 20:173–175.
6	Jefferson T, Demicheli V (1998) No evidence that vaccines cause insulin dependent diabetes mellitus. J Epidemiol Community Health 52:674–675.
7	THE INSTITUTE FOR VACCINE SAFETY DIABETES WORKSHOP PANEL (1999) Childhood immunizations and type 1 diabetes: summary of an Institute for Vaccine Safety Workshop. Pediatr Infect Dis J 18:217–222.
8	Classen JB, Classen DC (1999) Association between type 1 diabetes and hib vaccine. Causal relation is likely. BMJ 319:1133.
9	Elliman D (1999) Vaccination and type 1 diabetes mellitus. BMJ 318:1159–1160.
10	Classen JB, Classen DC (1999) Immunization in the First Month of Life may Explain Decline in Incidence of IDDM in the Netherlands. Autoimmunity 31:43–45.
11	Classen JB, Classen DC (2000) Hemophilus vaccine associated with increased risk of diabetes: causality likely. Diabetes care, 23:872-873.
12	Galama JM (2000) Vaccinations as risk factors for Type I diabetes mellitus. Diabetologia 43:684.
13	Milne LM (2000) Difficulties in assessing the relationship, if any, between mumps vaccination and diabetes mellitus in childhood. Vaccine 19:1018–1025.
14	Hummel M, Füchtenbusch M, Schenker M, Ziegler AG (2000) No major association of breast-feeding, vaccinations, and childhood viral diseases with early islet autoimmunity in the German BABYDIAB Study. Diabetes Care 23:969–974.
15	Chen RT, Pless R, Destefano F (2001) Epidemiology of autoimmune reactions induced by vaccination. J Autoimmun 16:309–318.

ESM Table 2 *continued*: List of 33 articles excluded from review after full-text screening.

	**Excluded papers**
16	Classen JB, Classen DC (2001) Vaccines and the risk of insulin-dependent diabetes (IDDM): potential mechanism of action. Med Hypotheses 57:532–538.
17	Robles DT, Eisenbarth GS (2001) Type 1A diabetes induced by infection and immunization. J Autoimmun 16:355–362.
18	Buschard K, Funda DP (2002) Vaccination studies in type 1 diabetes. Lancet 360:488; author reply 488.
19	Soltész G (2003) Diabetes in the young: a paediatric and epidemiological perspective. Diabetologia 46:447–454.
20	Sipetic S, Vlajinac H, Kocev N, Radmanovic S (2003) The belgrade childhood diabetes study: association of infections and vaccinations on diabetes in childhood. Ann Epidemiol 13:645–651.
21	Wasfy JH (2004) Childhood vaccination and type 1 diabetes. N Engl J Med 351:298–300.
22	Rubinstein E (2004) Vaccination and autoimmune diseases: the argument against. Isr Med Assoc J 6:433–435.
23	Tishler M, Shoenfeld Y (2004) Vaccination may be associated with autoimmune diseases. Isr Med Assoc J 6:430–432.
24	Classen JB (2004) Pertussis infections, vaccines and Type 1 diabetes. Diabet Med 21:397–398; author reply 398–399.
25	Demicheli V, Jefferson T, Rivetti A, Price D (2005) Vaccines for measles, mumps and rubella in children. Cochrane database Syst Rev 4.
26	Huppmann M, Baumgarten A, Ziegler A-G, Bonifacio E (2005) Neonatal Bacille Calmette-Guerin Vaccination and Type 1 Diabetes. Diabetes Care 28:1204–1206.
27	Sipetić SB, Vlajinac HD, Kocev NI, et al. (2005) The Belgrade childhood diabetes study: a multivariate analysis of risk determinants for diabetes. Eur J Public Health 15:117–122.
28	Rousseau M-C, Parent M-E, St-Pierre Y (2008) Potential health effects from non-specific stimulation of the immune function in early age: The example of BCG vaccination. Pediatr Allergy Immunol 19:438–448.
29	Silfverdal S-A, Nilsson L, Blennow M, et al. (2010) Vaccination of children - summary and conclusions from a systematic review. Acta Paediatr 99:1287–1289.

ESM Table 2 *continued*: List of 33 articles excluded from review after full-text screening.

	**Excluded papers**
30	Rasmussen TA, Jørgensen MRS, Bjerrum S, et al. (2012) Use of population based background rates of disease to assess vaccine safety in childhood and mass immunisation in Denmark: nationwide population based cohort study. BMJ 345:e5823.
31	Duderstadt SK, Rose CE, Real TM, et al. (2012) Vaccination and risk of type 1 diabetes mellitus in active component U.S. Military, 2002-2008. Vaccine 30:813–819.
32	Ovetchkine P Effets secondaires des vaccinations. Arch pédiatrie 8:316–320.
33	Vial T, Descotes J Autoimmune diseases and vaccinations. Eur J Dermatol 14:86–90.
