# Supplementary material for: Vaccinations and childhood type 1 diabetes mellitus: a meta-analysis of observational studies
Source: Diabetologia. 2015 Nov 12;59:237–43. doi: 10.1007/s00125-015-3800-8 (PMC4705121; doi:10.1007/s00125-015-3800-8)
Supplement: Supplementary file 5 — (PDF 132 kb) [file 125_2015_3800_MOESM5_ESM.pdf]

ESM Table 3: Vaccinations reported by studies, ordered by year of publication

**First author, year [reference]**	**Measles**	**Rubella**	**Mumps**	**Pertussis**	**BCG**	**HiB**	**Tetanus**	**Diphtheria**	**Poliomyelitis**	**MMR**	**DTP**	**H1N1**	**Tick-borne encephalitis**	**Hepatitis B**	**Meningitis C**	**Smallpox**
Glatthaar, 1988 [21]	✓	✓	-	✓	-	-	✓	✓	-	-	✓ [c]	-	-	-	-	-
Blom, 1991 [12]	✓	✓	✓	-	✓	-	✓	-	✓	-	-	-	-	-	-	✓
Telahun, 1994 [22]	✓	-	-	✓	✓	-	✓	✓	✓	-	✓ [c]	-	-	-	-	-
Verge, 1994 [20]	-	-	✓	-	-	-	-	-	-	-	-	-	-	-	-	-
Parent, 1997[a] [13]																
Series A	-	-	-	-	✓	-	-	-	-	-	-	-	-	-	-	-
Series B	-	-	-	-	✓	-	-	-	-	-	-	-	-	-	-	-
Karvonen, 1999[b] [14]																
Cohort 2	-	-	-	-	-	✓	-	-	-	-	-	-	-	-	-	-
Cohort 3	-	-	-	-	-	✓	-	-	-	-	-	-	-	-	-	-
Rami, 1999 [15]	✓	✓	✓	✓	✓	✓	✓	✓	✓	-	-	-	✓	-	-	-
EURODIAB Latvia, 2000 [6]	✓	✓	✓	✓	✓	-	✓	✓	✓	-	-	-	-	-	-	-
EURODIAB Lithuania, 2000 [6]	✓	✓	✓	✓	✓	-	✓	✓	✓	-	-	-	-	-	-	-
EURODIAB Luxembourg, 2000 [6]	✓	✓	✓	✓	✓	✓	✓	✓	✓	-	-	-	-	-	-	-
EURODIAB Romania, 2000 [6]	✓	✓	-	✓	-	✓	-	-	-	-	-	-	-	-	-	-
EURODIAB UK Yorkshire, 2000 [6]	✓	✓	✓	✓	✓	✓	✓	✓	✓	-	-	-	-	-	-	-
EURODIAB UK NI, 2000 [6]	✓	✓	✓	✓	✓	✓	✓	✓	✓	-	-	-	-	-	-	-
DeStefano, 2001 [16]	✓	✓	✓	✓	-	✓	-	-	-	✓ [c]	-	-	-	✓	-	-
Black, 2002 [18]	-	-	-	-	-	✓	-	-	-	-	-	-	-	-	-	-
Montgomery, 2002 [17]	-	-	-	✓	-	-	-	-	-	-	-	-	-	-	-	-
Altobelli, 2003 [23]	✓	✓	✓	✓	-	-	-	-	-	✓ [c]	-	-	-	-	-	-
Hviid, 2004 [5]	✓	✓	✓	-	-	✓	-	-	-	✓ [c]	-	-	-	-	-	-
Cardwell, 2008 [24]	✓	✓	✓	✓	✓	✓	-	-	-	✓ [c]	-	-	-	-	✓	-
Karavanaki, 2008 [19]	✓	✓	✓	-	-	-	-	-	-	✓ [c]	-	-	-	-	-	-
Skrodeniene, 2010 [25]	-	-	-	-	-	-	✓	✓	✓	-	✓ [c]	-	-	-	-	-
Bardage, 2011 [26]	-	-	-	-	-	-	-	-	-	-	-	✓	-	-	-	-

BCG, Bacillus Calmette–Guérin; HiB, haemophilus influenza B; MMR, mumps, measles, rubella; NI, Northern Ireland. [a]Estimates for two case-control series were provided by this article. [b]Estimates were provided in two different exposed groups compared to the same unexposed group. [c]These estimates were used both for combined vaccine and single vaccines.
